# Tumor–neuronal hybrid programs reveal a spatially adaptive malignant state in glioblastoma

**DOI:** 10.3389/fonc.2026.1865999

**Published:** 2026-08-03

**Authors:** Claire Yuan, Jake Y. Chen

**Affiliations:** 1 AlphaMind Club, Irvine, CA, United States; 2 Systems Pharmacology AI Research Center, School of Medicine, The University of Alabama at Birmingham, Birmingham, AL, United States

**Keywords:** cancer neuroscience, cellular plasticity, glioblastoma, neuronal mimicry, spatial transcriptomics, tumor-neuron interactions

## Abstract

**Objective:**

Glioblastoma (GBM) exhibits marked cellular plasticity, including partial acquisition of neuron-associated transcriptional features. We tested whether public GBM spatial and single-nucleus datasets reveal a tumor-neuronal program with spatial organization and distinction from canonical proneural-like identity.

**Methods:**

We re-analyzed public multi-region GBM spatial transcriptomic data from Greenwald et al. and used healthy cortex data from Ravi et al. as a non-malignant reference. Tumor-neuronal (TN) classifications were defined by concurrent enrichment of malignancy-associated and neuronal-associated gene programs using permutation-derived empirical thresholds. Classification stability was assessed by bootstrap resampling, repeated threshold estimation, and Jaccard overlap. Greenwald-derived CNA/malignancy scores were used to evaluate tumor-associated signal in TN-classified spatial units. External validation was performed in an independent Wang malignant GBM-cell/single-nucleus dataset, including held-out TN-specific scoring after excluding genes used in the original scoring programs. Exploratory bulk-cohort analysis was performed in TCGA GBM.

**Results:**

TN-classified spatial units were detected across tumor regions at higher frequency than healthy cortex and showed regional organization within the spatial discovery specimen. They expressed synaptic and vesicle-associated genes, including SYT1 and STMN2, while retaining malignant/glial-associated features such as GFAP, VIM, and CLU. Mature neuronal subtype markers were less enriched, supporting partial neuronal mimicry rather than lineage conversion. TN-associated genes formed a connected protein-interaction network centered on synaptic, neurite-associated, and cytoskeletal proteins. CNA/malignancy-score analysis showed tumor-associated signal in TN-classified spatial units. In the Wang malignant GBM-cell validation dataset, TN-classified cells/nuclei retained held-out TN-specific enrichment relative to non-TN and proneural-high non-TN malignant cells/nuclei.

**Conclusion:**

Public spatial and single-nucleus GBM datasets identify a tumor-associated neuronal mimicry program that is spatially organized in a GBM discovery specimen and detectable in an independent malignant-cell validation dataset. This program is proneural-adjacent but not reducible to canonical proneural identity.

## 1 Introduction

Glioblastoma (GBM) almost invariably recurs despite aggressive multimodality therapy, and survival remains poor despite maximal safe resection followed by radiotherapy and temozolomide (1, 2). This behavior is widely attributed to profound cellular plasticity, whereby GBM cells transition among alternative phenotypic states that support invasion, treatment evasion, and adaptation to the local microenvironment (3–5). One manifestation of this broader plasticity is neuronal mimicry, in which malignant cells acquire selected neuron-associated features without necessarily undergoing complete neuronal lineage conversion (6).

Tumor-neuron communication is now recognized as a major component of glioma biology. Neuronal activity can promote glioma growth through activity-dependent signaling, including neuroligin-3 release (7), and direct neuron-to-glioma synaptic and electrochemical interactions have been demonstrated in human tumors and experimental models (8, 9). Subsequent work showed that glioblastoma can hijack neuronal mechanisms to support brain invasion and that glioma synapses recruit adaptive plasticity programs linked to tumor progression (10, 11). In patients, functionally connected glioblastoma regions have been associated with worse survival and impaired cognition, reinforcing the clinical significance of tumor-neural circuit interactions (12). Together, these findings place cancer neuroscience at the center of contemporary GBM biology (13).

These biological advances were paralleled by single-cell and multi-omic studies showing that malignant GBM populations can express developmental, glial, and neuron-associated programs. Early single-cell RNA-seq work highlighted marked intratumoral heterogeneity in primary glioblastoma (14), while lineage and fate-mapping analyses supported neurodevelopmental hierarchies within malignant populations (15, 16). Subsequent state models resolved recurrent neural-progenitor-like, oligodendrocyte-progenitor-like, astrocyte-like, mesenchymal-like, and injury-response programs, emphasizing that GBM states are dynamic and context dependent (3–5, 16). Spatially resolved and multi-layered atlases have further shown that malignant ecosystems are organized by local niches, state proximities, and anatomical context, reinforcing the idea that GBM transcriptional states are shaped by tissue geography as well as cell-intrinsic programs (17–21).

Single-cell and single-nucleus sequencing resolve malignant-cell heterogeneity but lose tissue architecture, whereas bulk profiling averages across spatially distinct niches. Spatial transcriptomics preserves anatomical context and enables tumor-neuronal programs to be evaluated *in situ*, particularly when paired with malignant-cell or malignant-nucleus validation (17–21). In spot-based platforms such as 10x Genomics Visium, a spatial unit may contain multiple cells and extracellular RNA contributions. Therefore, concurrent tumor-associated and neuronal-associated transcriptional signal in a spatial unit may reflect a tumor-associated hybrid-like program, local admixture of malignant and neuronal elements, or both. For this reason, the present study reports Greenwald classifications as spatial-unit classifications and uses independent malignant-cell/single-nucleus data to test whether a related dual-program state is detectable at cellular resolution.

To address these questions, we characterized neuronal mimicry in GBM using a spatially resolved dual-program framework. We hypothesized that, if neuronal mimicry represents an adaptive tumor-associated state, TN-classified spatial units should: (1) show concurrent enrichment of malignant and neuronal-associated programs; (2) exhibit non-random spatial distribution within tumor regions; (3) carry tumor-associated CNA/malignancy signal; and (4) display reproducible gene-expression features consistent with a coordinated neuron-associated program. We then evaluated whether this framework transferred to an independent malignant GBM-cell/single-nucleus dataset and whether TN-classified malignant cells/nuclei retained features beyond the original scoring genes.

This study does not claim that neuronal-like GBM states are entirely new or that spot-level spatial classifications alone prove the existence of discrete tumor-neuronal hybrid cells. Instead, it tests whether a permutation-threshold dual-program framework identifies a tumor-associated neuronal mimicry program with spatial organization, tumor-associated signal, and distinction from canonical proneural identity. These questions intersect with established glioblastoma subtype frameworks, including classical, mesenchymal, and proneural classifications (22), as well as more recent single-cell state models emphasizing dynamic and context-dependent transcriptional programs (3–5, 16). Whether neuronal mimicry represents a variant of these canonical states, particularly the proneural program, or a distinct hybrid-like configuration remains unresolved.

## 2 Materials and methods

### 2.1 Data sources and preprocessing

We re-analyzed publicly available spatial transcriptomic, single-nucleus/single-cell, and bulk transcriptomic datasets from GBM and non-malignant human cortex. Dataset-level sample counts, analyzed-unit numbers, accession identifiers, platforms, and analytic roles are provided in **Supplementary Table 1**.

GBM spatial transcriptomic data were obtained from Greenwald et al. (18), which profiled multi-regional glioblastoma samples using integrative spatial transcriptomics, including 10x Genomics Visium, and matched CODEX proteomics. For the primary spatial discovery analysis, we focused on one GBM specimen that included three anatomically defined tumor regions collected via navigation-guided surgery: tumor core, bulk/non-core tumor, and infiltrative margin. This specimen was selected because it allowed region-aware analysis of tumor core, non-core tumor, and brain-interfacing infiltrative tissue within a shared spatial framework.

As a non-malignant reference, we included normal cortical data from Ravi et al. (17), which provided spatially resolved multi-omic maps of normal human cortex. Healthy cortex was used to establish baseline neuronal versus non-neuronal expression patterns in non-malignant tissue and was not treated as a patient-matched control for tumor-adjacent brain.

For spatial transcriptomics, we used processed gene-expression matrices linked to spatial coordinates and source-provided region annotations. In the Greenwald analysis, class labels are reported as spatial-unit classifications. Expression values were normalized and log-transformed according to the available processed-data workflow. No additional batch correction was applied within the primary GBM discovery specimen. Healthy reference data were processed in a compatible manner to support cross-dataset comparison.

Matched CODEX spatial proteomic data from the Greenwald GBM resource (18) were used to document availability of neuronal and tumor/glial-associated protein markers in the source dataset. CODEX data are treated as a resource annotation rather than as a primary protein-level validation layer in the present manuscript. The CODEX antibody panel is provided in **Supplementary Table 7**.

### 2.2 Spatial-unit scoring and TN classification

To identify tumor-neuronal program enrichment, we defined two gene programs: a malignancy program and a neuronal program. The neuronal gene list used for scoring is provided in **Supplementary Table 5**, and the malignant/tumor gene list used for scoring is provided in **Supplementary Table 6**. The malignancy program comprised genes associated with malignant glioma identity, including canonical GBM markers and genes linked to proliferative, stem-like, or tumor-associated states. The neuronal program comprised genes associated with neuronal identity and neuron-associated structure and signaling, including synaptic and neurite-related genes. Program gene lists were defined *a priori* from lineage-relevant and literature-supported markers and then applied unchanged across discovery and validation datasets.

Each spatial unit or cell/nucleus was scored for both programs using https://scanpy.readthedocs.io/en/stable/generated/scanpy.tl.score_genes.html (23), yielding a malignancy score and a neuronal score. To define empirical thresholds for program positivity, we used a permutation-based framework. For each program, 1,000 random control gene sets were generated from the detected-gene universe. Control gene sets were matched to the original program by gene-set size and approximate average-expression distribution using expression bins. Each control gene set was scored using the same procedure as the true program. The 99th percentile of the resulting null score distribution was used as the empirical threshold for significant enrichment of that program. Thresholds were estimated within each dataset to account for dataset-specific expression distributions and detection characteristics.

Observations exceeding the malignancy threshold were classified as malignancy-positive, and observations exceeding the neuronal threshold were classified as neuronal-positive. Observations exceeding both thresholds were classified as Tumor-neuronal (TN), those exceeding only the malignancy threshold as Tumor-program, those exceeding only the neuronal threshold as Neuronal-program, and those exceeding neither threshold as Other.

This procedure was designed to reduce arbitrariness in threshold selection and to distinguish reproducible dual-program enrichment from isolated expression of individual genes. Because hybrid-like state assignment could depend on gene-set composition or thresholding, we evaluated robustness empirically through repeated null-threshold estimation, bootstrap resampling, and concordance analysis. Robustness was summarized using Jaccard overlap across repeated classifications and by evaluating class stability under repeated threshold estimation.

Throughout the manuscript, “class” refers to the mutually exclusive categories Tumor-program, Neuronal-program, TN, and Other, assigned by the dual-program permutation-threshold framework. In Greenwald spatial analyses, class labels refer to spatial units and should not be interpreted as single-cell identities. In Wang validation analyses, downstream interpretation focuses on TN-classified malignant cells/nuclei, Tumor-program malignant cells/nuclei, non-TN malignant cells/nuclei, and proneural-high non-TN malignant cells/nuclei.

### 2.3 Distinguishing TN classification from spatial admixture

Because spot-based spatial transcriptomic platforms may capture multiple cells per spatial unit, we evaluated whether TN-classified spatial units reflected tumor-associated neuronal mimicry rather than normal neuronal signal alone. First, TN spatial-unit frequencies were compared with healthy cortex, where neuronal program enrichment should be common but malignant program enrichment should be low. Healthy cortex was dominated by Neuronal-program and Other classifications and showed a lower TN fraction than tumor regions.

Second, Greenwald-derived CNA/malignancy scores were integrated with TN classifications to test whether TN-classified spatial units carried tumor-associated signal. TN-classified units with higher CNA/malignancy scores than lower-malignancy spatial classes were interpreted as supporting tumor association.

Third, the same scoring framework was transferred to an independent Wang malignant GBM-cell/single-nucleus dataset. In that dataset, TN classifications were evaluated within the malignant GBM-cell subset, providing a cellular-resolution validation layer less susceptible to spot-level admixture. Held-out TN-specific scoring after exclusion of original neuronal and malignancy scoring genes was used to evaluate TN-associated signal beyond the original classification genes.

### 2.4 Differential expression and signature analyses

To define the molecular profile of TN-classified spatial units, we first performed differential-expression analyses comparing TN-classified units with non-TN units within each tumor region. Unless otherwise stated, the non-TN comparator included all non-TN spatial units within the same region. Region-stratified analysis was used to reduce confounding by baseline compartment differences. Differential expression was assessed using Wilcoxon rank-sum testing as implemented in Scanpy (23).

The highest-ranked TN-upregulated genes within each region are provided in **Supplementary Table 2**. Ranking was based on statistical significance, effect size, and direction of enrichment in TN-classified spatial units. This table reports the strongest TN-upregulated genes in Core, bulk/non-core tumor, and Infiltrative regions.

As a complementary secondary analysis, we used a one-versus-rest differential-expression framework across the permutation-defined classes to identify genes enriched in each class relative to all remaining classes combined. Genes upregulated in TN-classified observations relative to Tumor-program, Neuronal-program, and Other observations were used to define a TN-enriched transcriptional signature for exploratory interpretation and bulk-cohort scoring.

As a secondary comparison focused on proneural context, we defined a proneural-high non-TN reference population as non-TN observations in the upper decile of proneural score and compared these observations directly with TN-classified observations to test whether the TN state could be reduced to a canonical proneural-like program. Where possible, TN-associated features were also compared against established GBM single-cell state programs, including proneural/neural-progenitor-like, oligodendrocyte-progenitor-like, astrocyte-like, mesenchymal-like, cell-cycle, stress, and hypoxia-associated programs.

### 2.5 Protein-protein interaction analysis

To assess whether TN-upregulated genes formed a biologically related program rather than a disconnected set of markers, we performed protein-protein interaction analysis using STRING v12 (24). We examined observed and expected edge counts, average node degree, average local clustering coefficient, PPI enrichment relative to STRING random-network expectations, network topology, and the presence of highly connected nodes.

### 2.6 External malignant-cell validation in Wang et al.

To evaluate generalizability beyond the discovery specimen, we applied the same scoring and classification pipeline to an independent malignant GBM-cell/single-nucleus RNA-seq dataset from Wang et al. (19), which profiled paired tumor core and peritumoral brain tissues from patients with GBM. The same malignancy and neuronal programs and the same empirical thresholding strategy were used without modification. The validation analysis was performed in the malignant GBM-cell subset available in the processed object used for this study, comprising 34,023 GBM cells/nuclei from four patients.

Because neuronal and malignancy program scores contribute to TN classification, their distributions in the Wang dataset were interpreted as transfer of the dual-program classification framework rather than as independent validation. To add a held-out validation layer, all genes present in the original neuronal and malignancy scoring programs were removed from downstream validation gene sets. We then computed held-out TN-associated and TN-specific scores using the remaining genes with https://scanpy.readthedocs.io/en/stable/generated/scanpy.tl.score_genes.html (23). Scores were compared between TN-classified malignant cells/nuclei and non-TN malignant cells/nuclei, between TN-classified and Tumor-program malignant cells/nuclei, and between TN-classified and proneural-high non-TN malignant cells/nuclei. Pairwise comparisons were performed using Mann-Whitney U tests with Benjamini-Hochberg correction, and multi-group comparisons were assessed using Kruskal-Wallis tests where applicable.

To reduce the risk that pooled validation results were driven by one patient or sample, class counts and held-out score distributions were summarized by patient/sample where metadata permitted. These analyses are reported in **Supplementary Table 4**.

### 2.7 CNA/malignancy-score validation of spatial TN units

To evaluate tumor-associated signals in TN-classified spatial units, we integrated Greenwald-derived CNA/malignancy scores with our spatial-unit classifications. CNA/malignancy scores were matched to spatial barcodes within the Core, bulk/non-core tumor, and Infiltrative Greenwald tumor regions and compared across Tumor-program, TN, Neuronal-program, and Other classes. CNA/malignancy-score distributions were summarized by region and class, and pairwise comparisons were performed using Wilcoxon/Mann-Whitney rank-sum tests with Benjamini-Hochberg correction. The corresponding distributions are shown in **Supplementary Figure 1**, and class-level summary statistics are provided in **Supplementary Table 3**.

### 2.8 Exploratory bulk-cohort analysis

As a secondary exploratory analysis, we derived a TN-enriched score from genes selectively upregulated in TN-classified observations relative to all other classes in the one-versus-rest analysis. This score reflects enrichment of the TN-enriched transcriptional program rather than direct measurement of TN-cell abundance. TN-enriched scores were calculated across TCGA GBM bulk transcriptomic data accessed through UCSC Xena (25) and used to stratify patients for exploratory survival analysis.

Patients in the top 20% and bottom 20% of score values were compared using the log-rank test. Sample numbers were reported for the full matched cohort and for each survival stratum. A univariable Cox proportional-hazards model was also fit using continuous TNscore. TCGA results were interpreted as exploratory cohort-level associations because bulk GBM survival associations may be influenced by clinical and molecular variables including age, treatment, tumor purity, MGMT promoter methylation, IDH status, and transcriptional subtype.

### 2.9 Statistical analysis

All statistical analyses were performed using Python with Scanpy (23), SciPy (26), and statsmodels (27), and R for selected enrichment analyses. Differential-expression p-values were adjusted for multiple testing using the Benjamini-Hochberg procedure. Adjusted p < 0.05 was considered significant unless otherwise specified. Enrichment analyses were similarly corrected for multiple comparisons. For comparisons of class frequencies across regions, observed class fractions and pairwise or region-level statistical tests were reported as appropriate; interpretation was based on both enrichment magnitude and observed regional context.

We did not assume independence between malignancy and neuronal module scores. Accordingly, robustness of TN assignment was evaluated empirically through bootstrap resampling, repeated null-threshold estimation, and Jaccard overlap across repeated runs. Sensitivity analyses using alternative empirical thresholds and modified gene-list compositions were used where available to evaluate classification stability.

## 3 Results

### 3.1 Spatial identification of tumor-neuronal program enrichment in a GBM discovery specimen

**Figure 1:**
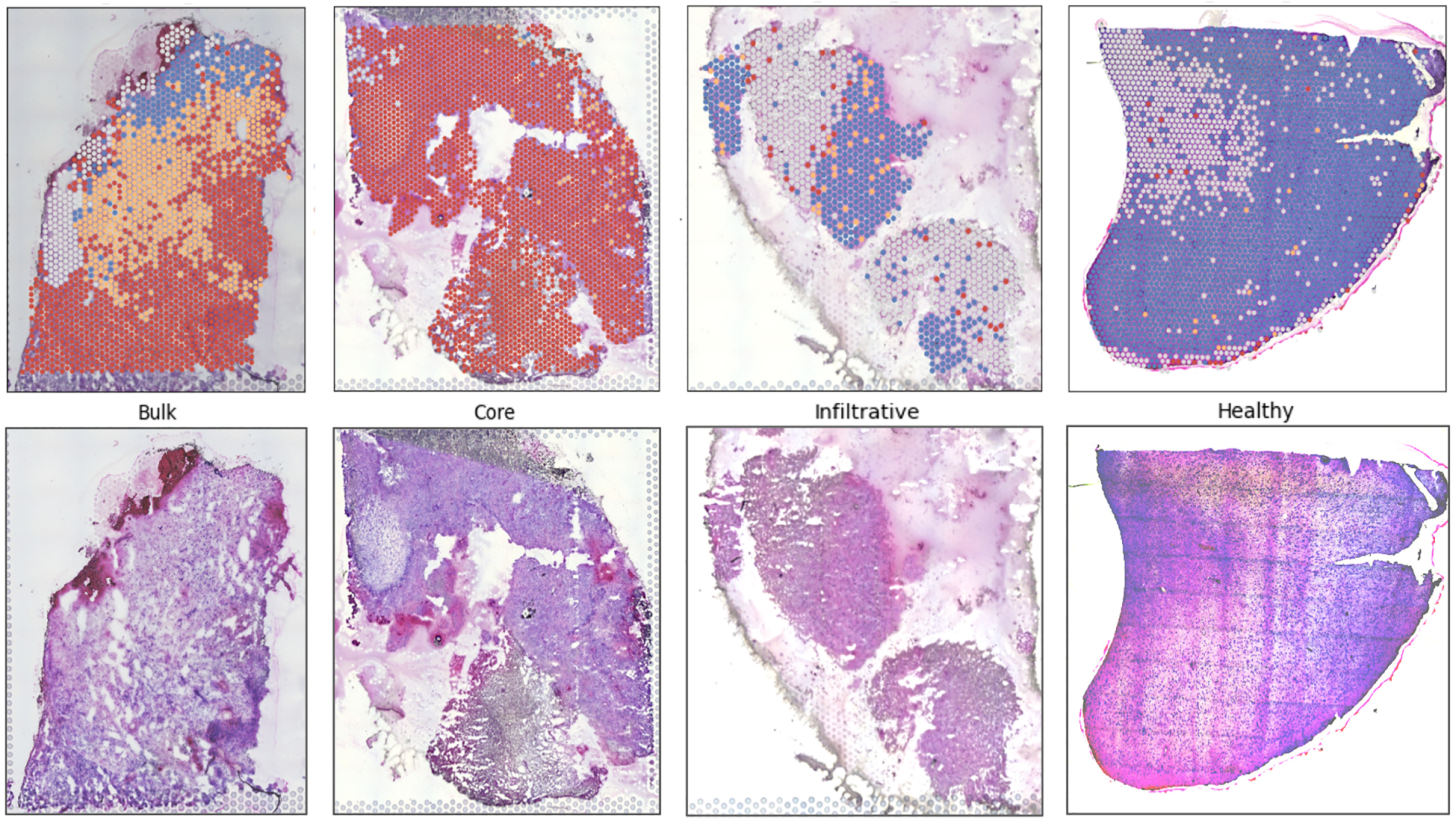
Histologic context and spatial distribution of tumor, neuronal-program, and tumor-neuronal program-enriched spatial units in glioblastoma. H&E histology images and corresponding spatial classification maps are shown for healthy cortex, tumor core, infiltrative margin, and bulk/non-core tumor regions. Spatial units were assigned to tumor-program, neuronal-program, TN, or other categories using a permutation-based dual-program classification approach. Histology images provide tissue context for the adjacent spot-level spatial maps. Region-specific patterns are observed, with neuronal-program units predominating in healthy cortex, tumor-program units dominating the core, and TN-classified units detected across tumor regions.

Using integrated spatial transcriptomics, we mapped tumor, neuronal, and dual-program-enriched spatial units across the GBM discovery specimen. **Figure 1** shows H&E histology images and corresponding spatial classification maps from Core, bulk/non-core tumor, Infiltrative, and Healthy cortex, with spatial units colored by class assignment: Tumor-program, Neuronal-program, TN, or Other.

Marked regional differences were observed within the discovery specimen. In the tumor core, most spatial units were classified as Tumor-program, consistent with a densely malignant compartment. TN-classified spatial units were nevertheless detected in the core. The bulk/non-core tumor region contained a mixture of Tumor-program, TN, Neuronal-program, and Other spatial units. The Infiltrative region also showed TN-classified spatial units intermingled with Neuronal-program and Other populations, consistent with spatial complexity at the tumor-brain interface. In Healthy cortex, spatial units were dominated by Neuronal-program and Other classifications and showed a lower TN fraction than tumor regions.

To quantify these patterns, we compared class frequencies across regions. TN-classified spatial units were detected across tumor regions and occurred at higher frequency in tumor-region tissue than in the healthy cortex reference. The discovery specimen therefore showed regional organization of dual tumor-neuronal program enrichment, rather than random distribution across sampled tissue contexts. Regional class composition is summarized in **Supplementary Figure 2**.

Because Greenwald uses spot-based spatial transcriptomics, TN classifications are interpreted as spatial-unit enrichments rather than definitive single-cell identities.

### 3.2 TN-classified spatial units exhibit a partial neuronal mimicry program

Differential-expression analysis identified genes upregulated in TN-classified spatial units relative to non-TN populations within tumor regions. Prominent TN-associated genes included SNAP25, SYT1, STMN2, and MAP2, indicating activation of a neuron-associated signature centered on vesicle handling, neurite organization, and cytoskeletal regulation.

At the same time, TN-classified units did not resemble fully differentiated neuronal subtypes. Canonical subtype-associated markers, including SLC17A7 and CCK, were less enriched relative to bona fide neurons. This pattern supports partial neuronal mimicry rather than full neuronal conversion, in which tumor-associated spatial units acquire selected neuron-associated features without complete neuronal subtype specification.

TN-classified units also retained malignant/glial-associated expression. Genes such as CLU, ACTB, GFAP, and VIM remained prominent in TN-classified units, indicating preservation of tumor/glial-associated identity. Absolute expression analysis further supported this interpretation: neuron-associated genes were meaningfully expressed alongside glioma-relevant transcripts. Together, these findings are consistent with a hybrid-like transcriptional configuration in which a neuron-associated module is superimposed on a persistent tumor-associated or glial-associated backbone. The highest-ranked TN-upregulated genes by region are provided in **Supplementary Table 2**.

### 3.3 TN-associated genes form a biologically related interaction network

**Figure 2:**
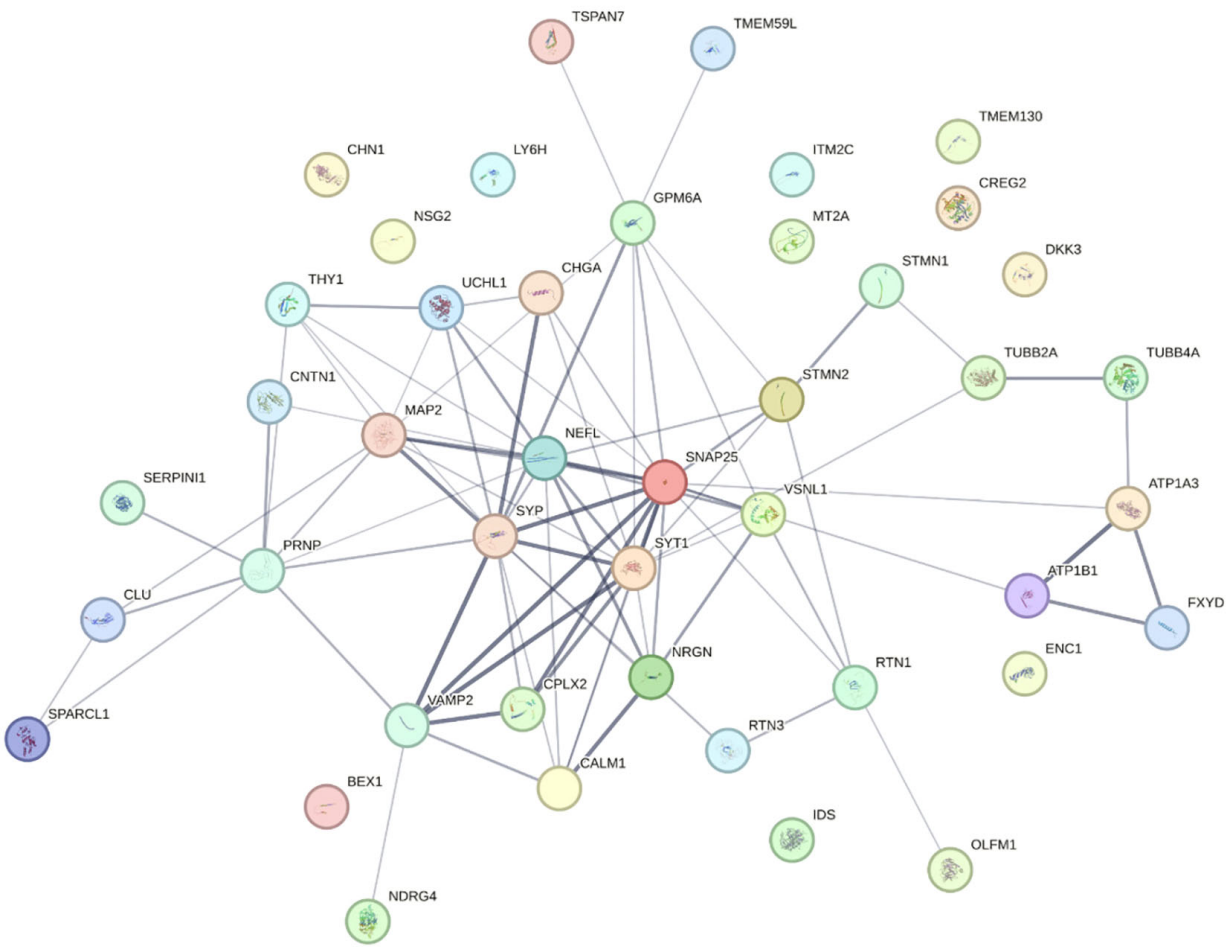
Protein-protein interaction network of tumor-neuronal signature genes. Network analysis of genes enriched in TN-classified observations identified a connected interaction module centered on synaptic and neurite-associated proteins. The network is enriched for functions related to synaptic vesicle cycling, axonal and neurite organization, and cytoskeletal remodeling. These findings support biological coherence among TN-associated genes.

We next asked whether the TN-associated signature reflected a coordinated biological program. PPI analysis showed that TN-upregulated genes formed a connected STRING interaction network (**Figure 2**). The network contained 43 nodes and 84 observed edges, compared with 8 expected edges under the STRING random-network model. The average node degree was 3.91, and the average local clustering coefficient was 0.407. The network showed strong PPI enrichment relative to random expectation, with STRING PPI enrichment p-value < 1.0 × 10^-16.

Prominent network genes included SNAP25, SYT1, NEFL, MAP2, and STMN2, and the network was centered on synaptic, neurite-associated, and cytoskeletal proteins. This network structure is consistent with biological relatedness among TN-associated genes and supports convergence on known neuronal structural and synaptic biology.

### 3.4 Complementary class-enriched analysis suggests additional TN features

As a secondary analysis, we examined whether TN-classified observations exhibit transcriptional features beyond overlap between malignant and neuronal programs. TN-classified observations showed enrichment of genes including BGN and MCAM, associated with extracellular matrix remodeling and cell adhesion; ID1 and ID3, regulators linked to plasticity and stem-like states; and APP and SCRG1, which have been implicated in neuronal or neurotrophic signaling contexts.

These findings suggest that TN-classified observations possess transcriptional characteristics beyond dual-program positivity alone. Highest-ranked regional TN-upregulated genes are provided in **Supplementary Table 2**.

### 3.5 TN is proneural-adjacent but not reducible to a canonical proneural program

Because TN-classified observations were enriched in proneural-like subtype contexts defined relative to established GBM subtype and state frameworks (3, 22), we next asked whether the TN state could be explained simply as a proneural program under a different label. To address this directly, we compared canonical proneural gene sets with TN-associated and TN-enriched signatures, evaluated observation-level relationships between proneural and TN scores, and performed a focused comparison between TN-classified observations and proneural-high non-TN observations.

**Figure 3:**
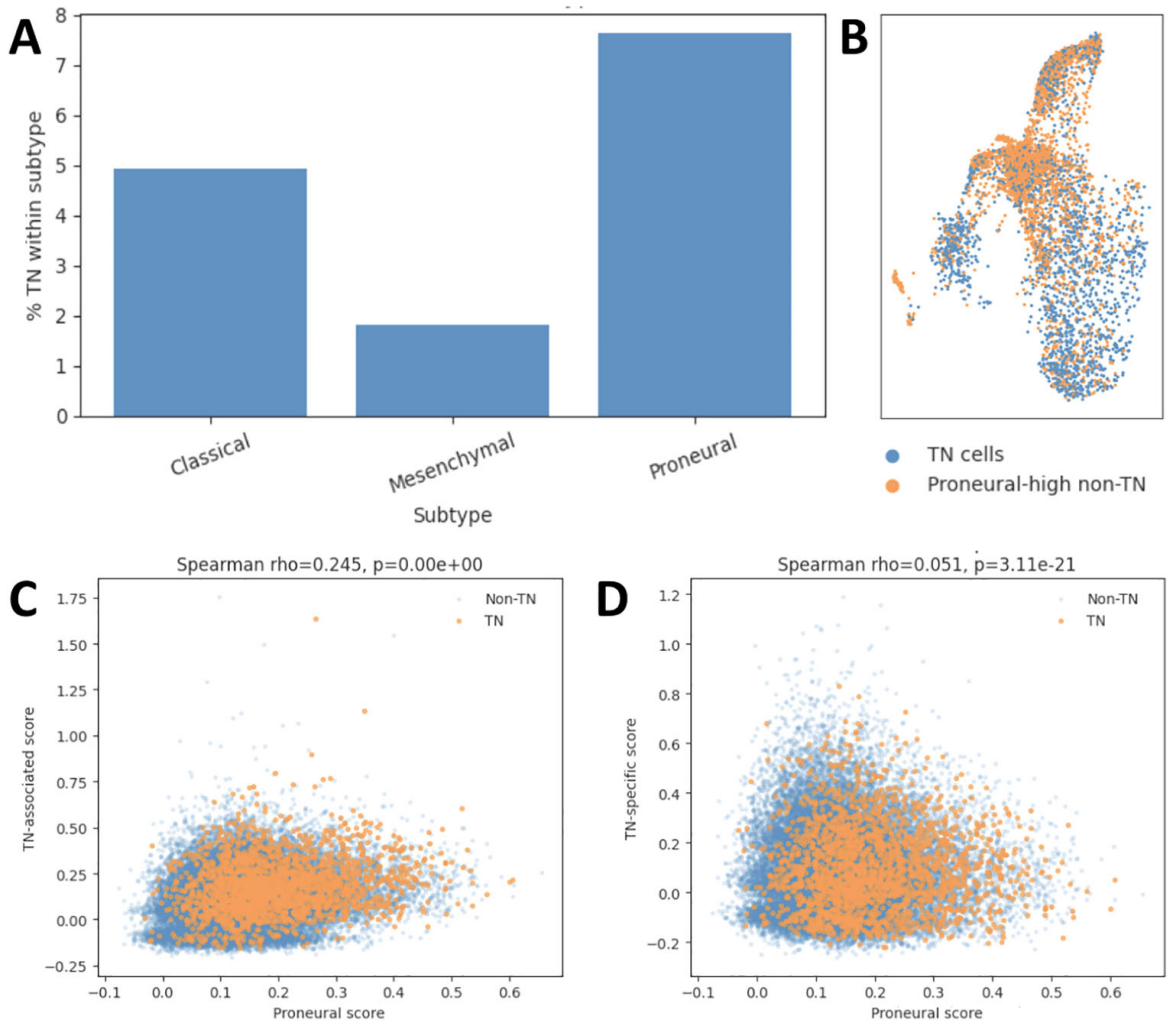
Tumor-neuronal observations are enriched in proneural contexts but are not equivalent to the proneural state. **(A)** TN-classified observations are most frequent in proneural-associated contexts, with lower representation in classical and mesenchymal contexts. **(B)** UMAP comparison of TN-classified observations and proneural-high non-TN observations shows partial overlap but incomplete concordance. **(C)** Correlation between proneural score and TN-associated score shows a modest positive relationship, indicating partial shared transcriptional features. **(D)** Correlation between proneural score and TN-specific score is weak, indicating that features most specific to the TN state are not explained by proneural identity alone. Together, these results support TN classification as proneural-adjacent but not reducible to canonical proneural identity.

**Figure 3A** shows that TN-classified observations were most frequent in proneural-associated contexts, although TN classification was not restricted to the proneural category. In low-dimensional expression space, TN-classified observations and proneural-high non-TN observations showed partial overlap but incomplete concordance (**Figure 3B**).

At the score level, proneural and TN scores showed limited concordance. The proneural score was weakly positively correlated with the TN-associated score, with Spearman rho = 0.245, but (**Figure 3C**) showed only negligible correlation with the TN-specific score, with (**Figure 3D**) rho = 0.051. Thus, while TN-associated features were partly aligned with proneural context, the TN-specific component was not meaningfully captured by proneural score alone.

To test this more stringently, we compared TN-classified observations against a non-TN reference restricted to the upper decile of proneural score. This comparison was designed to challenge the alternative explanation that TN-classified observations merely represent the most proneural-like subset of non-TN observations. Despite this stringent comparator, TN-classified observations remained transcriptionally distinct. TN-upregulated genes included neuronal and synaptic-associated features such as CNTNAP2, SCN1A, GRIA1, NRXN1, and SATB2, but also retained malignant, reactive, and interface-associated genes including GFAP, EGFR, CD44, VIM, FN1, TNC, F3, PTN, and VEGFA. In contrast, proneural-high non-TN observations were enriched for a comparatively cleaner neural-developmental program, including GRIA2, SOX4, CHD7, HES6, NCAM1, TNR, and DNM3.

Taken together, these analyses indicate that TN-classified observations arise in a partly proneural-adjacent context yet are not reducible to canonical proneural identity. Rather than representing a simple proneural-high state, TN-classified observations occupy a hybrid-like transcriptional state that combines neuron-associated features with persistent malignant, reactive, and microenvironment-adaptive programs.

### 3.6 CNA/malignancy-score validation supports tumor-associated signal in TN-classified spatial units

To evaluate whether TN-classified spatial units carried tumor-associated genomic signal, we integrated Greenwald-derived CNA/malignancy scores with our spatial-unit classifications. CNA/malignancy scores were available for 5,430 tumor-region spatial units. TN-classified spatial units showed intermediate CNA/malignancy scores, with a median score of 0.493 compared with 0.646 for Tumor-program units, 0.305 for Neuronal-program units, and 0.275 for Other units.

In pooled analysis, TN-classified spatial units had lower CNA/malignancy scores than Tumor-program units but higher scores than Neuronal-program and Other units. Pairwise tests showed significant differences between TN and Tumor-program units, TN and Neuronal-program units, and TN and Other units. Region-stratified distributions showed the same central pattern: TN-classified spatial units generally occupied an intermediate CNA/malignancy-score range between Tumor-program and lower-malignancy-score spatial classes.

These findings indicate tumor-associated CNA/malignancy signal in the TN spatial class and argue against an explanation based only on normal neuronal signal. The intermediate magnitude of CNA/malignancy scores is also compatible with the mixed tumor-brain microenvironment captured by spot-based spatial transcriptomics at the tumor-brain interface. Together with spatial organization and dual-program expression, the CNA/malignancy analysis identifies TN-classified spatial units as tumor-associated regions with concurrent neuronal-program enrichment. CNA/malignancy-score distributions are shown in **Supplementary Figure 1**, and summary statistics are provided in **Supplementary Table 3**.

### 3.7 External malignant-cell validation supports held-out TN-specific enrichment

To test whether the neuronal mimicry framework generalized beyond the primary spatial dataset, we applied the same scoring and classification pipeline to the independent Wang malignant GBM-cell/single-nucleus dataset. The validation subset contained 34,023 malignant GBM cells/nuclei from four patients. Downstream validation focused on TN-classified malignant cells/nuclei, Tumor-program malignant cells/nuclei, non-TN malignant cells/nuclei, and proneural-high non-TN malignant cells/nuclei.

TN-classified Wang GBM cells/nuclei retained malignancy-program signal while showing elevated neuronal-program scores relative to Tumor-program malignant cells/nuclei, supporting transfer of the dual-program framework to an independent malignant-cell dataset. Because the same neuronal and malignancy scores define TN classification, these score distributions were interpreted as framework transfer rather than independent validation.

To evaluate TN-associated signal beyond the original scoring genes, we computed held-out TN-specific scores after excluding all genes used in the original neuronal and malignancy scoring programs. TN-classified Wang GBM cells/nuclei showed higher held-out TN-specific scores than non-TN cells/nuclei overall and proneural-high non-TN cells/nuclei. TN-specific scores were not significantly different between TN-classified and Tumor-program cells/nuclei, consistent with TN occupying a tumor-adjacent malignant transcriptional state rather than a separate non-malignant neuronal population.

Together, these results indicate that the TN framework is not confined to the discovery spatial dataset and that TN-classified malignant GBM cells/nuclei occupy a tumor-adjacent transcriptional position with neuronal-program enrichment and held-out TN-specific signal. Full Wang validation statistics are provided in **Supplementary Table 4**.

### 3.8 Exploratory bulk-cohort analysis of a TN-enriched score

**Figure 4:**
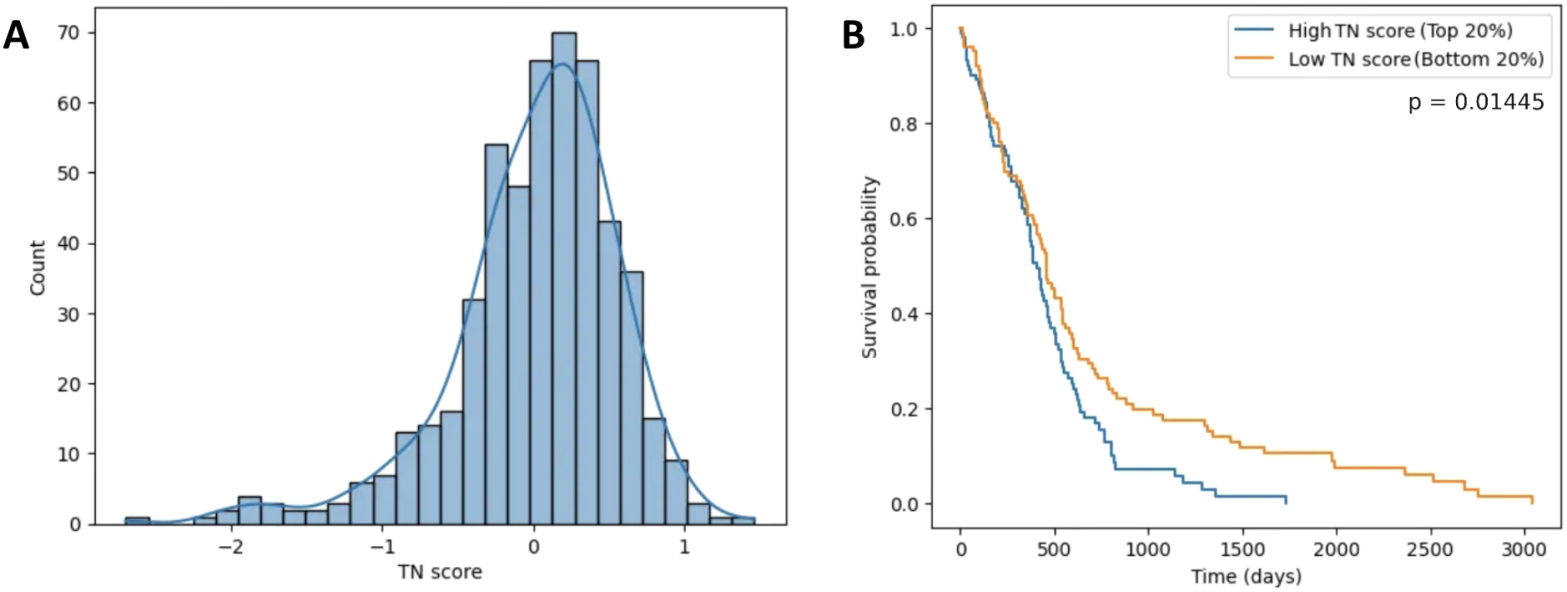
Exploratory survival analysis of the tumor-neuronal score in TCGA bulk glioblastoma. **(A)** Distribution of tumor-neuronal score values across TCGA GBM patients, showing inter-patient variability. **(B)** Kaplan-Meier analysis comparing patients in the top 20% and bottom 20% of the tumor-neuronal score distribution. The analysis included 518 matched TCGA GBM samples overall, with 104 samples in the top TNscore quintile and 104 samples in the bottom TNscore quintile. Higher tumor-neuronal scores were associated with poorer overall survival by log-rank testing, with test statistic = 5.9812 and p = 0.014459. Continuous TNscore showed a positive but non-significant trend in univariable Cox analysis. These findings suggest potential clinical relevance of the tumor-neuronal program but should be interpreted as exploratory because the analysis is based on bulk expression and was not adjusted for all major clinical and molecular covariates.

As a secondary exploratory analysis, we applied a TN-enriched score derived from the one-versus-rest analysis to TCGA GBM bulk transcriptomic data accessed through UCSC Xena. The analysis included 518 TCGA GBM samples with matched expression, overall survival time, and event status. Seventeen of the 20 TNscore genes were detected in the TCGA expression matrix. TN-enriched scores showed inter-patient variability, suggesting heterogeneity in expression of this signature across tumors (**Figure 4A**).

When patients in the top and bottom score quintiles were compared, tumors with high TN-enriched scores had shorter overall survival than tumors with low scores (**Figure 4B**). The top TNscore quintile contained 104 samples and the bottom TNscore quintile contained 104 samples. Kaplan-Meier analysis showed separation between the groups, with the high-score group displaying a steeper survival decline relative to the low-score group. The difference was significant by log-rank testing, with test statistic = 5.9812 and p = 0.014459.

To further assess the bulk-cohort association, we fit a univariable Cox proportional-hazards model using continuous TNscore. Continuous TNscore showed a positive but non-significant trend toward worse survival, with HR = 1.17, 95% CI = 0.985–1.392, and p = 0.0633.

This result suggests a possible bulk-tumor association between TN-enriched transcriptional features and adverse clinical behavior. Because the continuous Cox model did not reach conventional statistical significance and because the analysis was not adjusted for major clinical or molecular covariates, the TCGA result is interpreted as exploratory. The score reflects bulk expression of a TN-enriched program rather than direct measurement of TN-cell abundance or independent prognostic value.

## 4 Discussion

This study identifies a tumor-associated neuronal mimicry program in glioblastoma using spatial discovery analysis, CNA/malignancy-score support, and independent malignant-cell/single-nucleus validation. In the Greenwald spatial dataset, TN-classified spatial units were detected across tumor regions and showed regionally patterned localization within one GBM discovery specimen. These units expressed neuron-associated genes involved in synaptic, vesicle-associated, and neurite-related processes while retaining malignant/glial-associated features. CNA/malignancy-score analysis further indicated tumor-associated signals in TN-classified spatial units, arguing against an explanation based only on normal neuronal signal.

A central implication of these findings is that neuronal mimicry in GBM is better understood as partial acquisition of neuron-associated programs rather than full neuronal conversion. TN-classified observations retained tumor-associated expression while upregulating selected neuronal features yet did not show strong fidelity to mature neuronal subtype programs. This pattern supports a model of adaptive plasticity in which GBM cells selectively co-opt neuronal transcriptional modules without undergoing lineage conversion.

The TN program was also proneural-adjacent but not proneural-equivalent. Although TN-classified observations were enriched in proneural-associated contexts, proneural score showed limited concordance with TN-specific score, and TN-classified observations remained distinct from proneural-high non-TN comparators. In the Wang malignant GBM-cell validation dataset, TN-classified cells/nuclei retained held-out TN-specific enrichment after exclusion of genes used in the original neuronal and malignancy scoring programs. This supports the interpretation that TN classification captures features beyond the original dual-score definition and is not simply a relabeling of canonical proneural identity.

The class-enriched analyses suggest that the TN-enriched signature includes features linked to adhesion, extracellular matrix remodeling, and plasticity-related processes. These findings are consistent with a broader model in which neuronal mimicry is coupled to tissue interaction and niche adaptation at the tumor-brain boundary, where neuronal signaling, adaptive synaptic plasticity, and invasive developmental programs may converge (10, 11, 28, 29). The exploratory TCGA analysis further suggests that TN-enriched transcriptional features may track clinically relevant tumor biology, although bulk-tumor analyses do not directly measure TN-cell abundance and the observed survival association was not confirmed as an independent prognostic effect.

Several boundaries define the interpretation of the study. The primary spatial discovery analysis was performed in one GBM specimen selected for region-aware analysis, and Greenwald spatial classifications are based on spatial units rather than single cells. CNA/malignancy-score analysis and Wang malignant-cell validation support tumor association, while future larger spatial cohorts will be important for determining how consistently TN-classified spatial units appear across patients, molecular subtypes, and treatment contexts. The scoring framework depends on predefined neuronal and malignancy gene lists, although permutation thresholds, robustness analysis, and held-out validation were used to support stability. CODEX data were used to document marker availability in the source dataset and were not used here as formal protein-level validation. TCGA analysis was exploratory, based on bulk expression, and not adjusted for all clinically relevant covariates.

Future studies should validate this program using larger spatial cohorts, higher-resolution spatial transcriptomics, spatial proteogenomics, chromatin-level profiling, and functional perturbation experiments. Particularly important will be direct tests of whether TN-like malignant cells exhibit altered invasion, neuronal integration, synaptic responsiveness, therapy resistance, or niche adaptation at the tumor-brain interface.

Together, these findings position neuronal mimicry as a context-dependent axis of GBM plasticity. Rather than representing complete neuronal conversion, the TN program appears to reflect selective acquisition of neuron-associated features within a retained malignant state, particularly in spatial contexts where tumor cells interface with surrounding brain tissue.

## 5 Conclusion

Public GBM spatial and single-nucleus datasets identify a tumor-neuronal mimicry program that is spatially organized in a GBM discovery specimen and detectable in an independent malignant-cell validation dataset. TN-classified observations retain tumor-associated identity while selectively activating neuron-associated programs, supporting neuronal mimicry as a partial malignant plasticity state rather than full neuronal conversion.

This program is proneural-adjacent but not reducible to canonical proneural identity. Its association with adhesion, extracellular-matrix, and interface-related features suggests a potential role in local tissue adaptation at the tumor-brain boundary. Functional studies, larger spatial cohorts, and higher-resolution validation will be needed to determine whether this transcriptional state directly contributes to invasion, progression, neural integration, or therapeutic resistance.

## Data Availability

The original contributions presented in the study are included in the article/**Supplementary Material**. Further inquiries can be directed to the corresponding author.

## Supplementary material

Supplementary Figure 1 CNA/malignancy-score validation of TN-classified spatial units. CNA/malignancy-score distributions for Greenwald tumor-region spatial units, shown by dual-program spatial-unit class and by anatomical region. Panel A summarizes CNA/malignancy scores across Tumor-program, TN, Neuronal-program, and Other spatial units. Panel B shows region-stratified CNA/malignancy-score distributions across Core, bulk/non-core, and Infiltrative tumor regions.

Supplementary Figure 2 Regional composition of dual-program spatial-unit classes. Stacked bar plot showing the fraction of Tumor-program, Neuronal-program, TN, and Other spatial units across healthy cortex, tumor core, bulk/non-core tumor, and infiltrative regions. Class labels refer to spot-level spatial units rather than individual cells.

Supplementary Table 1 Dataset summary and analyzed-unit counts. Summary of datasets used in the study, including Ravi healthy cortex reference data, Greenwald GBM spatial tumor-region data, Wang malignant GBM-cell validation data, and the TCGA GBM exploratory bulk-expression cohort. The table includes dataset source, platform, number of patients/samples where available, number of spatial units or cells/nuclei analyzed, tissue region, and analytic purpose.

Supplementary Table 2 Differential expression results for TN-classified observations. Ranked region-stratified differential-expression results for TN-upregulated genes in Core, bulk/non-core, and Infiltrative tumor regions. Class labels refer to score-defined spatial-unit categories rather than single-cell identities.

Supplementary Table 3 CNA/malignancy-score validation of Greenwald spatial classifications. Class-level CNA/malignancy-score summary statistics for Greenwald tumor-region spatial units, including number of spatial units, mean score, median score, standard deviation, minimum, and maximum.

Supplementary Table 4 Wang malignant-cell validation and held-out gene-score analysis. Quantitative summary of Wang malignant GBM-cell validation analyses, including class/group comparisons, held-out TN-specific scoring after exclusion of original neuronal and malignancy scoring genes, statistical tests, adjusted p-values, and effect-size summaries.

Supplementary Table 5 Neuronal scoring gene list. *A priori* neuronal program gene list used for dual-program spatial-unit and malignant-cell scoring.

Supplementary Table 6 Malignancy scoring gene list. *A priori* malignant/tumor program gene list used for dual-program spatial-unit and malignant-cell scoring.

Supplementary Table 7 CODEX antibody-panel availability. Available neuronal, tumor/glial-associated, proliferation, and microenvironmental markers from the matched CODEX resource used to assess marker availability for orthogonal validation.
